# Supplementary figures and images for: A comprehensive study of SARS-CoV-2 main protease (Mpro) inhibitor-resistant mutants selected in a VSV-based system
Source: PLoS Pathog. 2024 Sep 11;20(9):e1012522. doi: 10.1371/journal.ppat.1012522 (PMC11407635; doi:10.1371/journal.ppat.1012522)

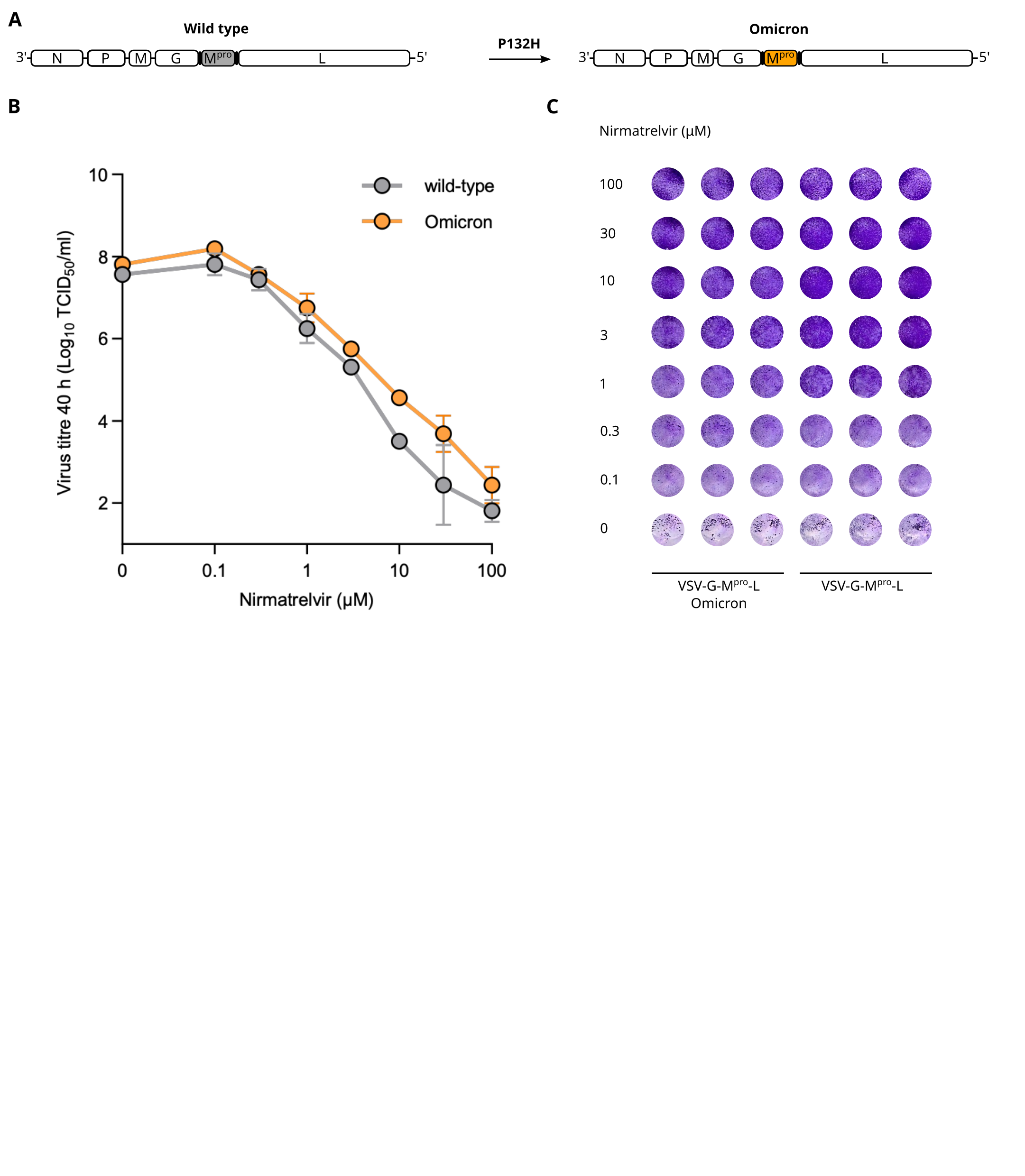

Supplement: S1 Fig — (A) Schematic representation of VSV-Mpro and VSV-Omicron-Mpro genomes. VSV-Omicron-Mpro was generated by introducing the substitution P to H at amino acid position 132 (P132H). (B) Dose response curves of VSV-Mpro and VSV-Omicron-Mpro against nirmatrelvir. Data are presented as geometric mean of n = 2 biologically independent replicates per condition. Each biological replicate consisted of n = 8 technical replicates. (C) Crystal violet staining of BHK21 cells used for dose response experiments with VSV-Mpro. Data are presented as the mean of n = 3 biologically independent replicates per condition. (TIF) [file ppat.1012522.s001.tif]

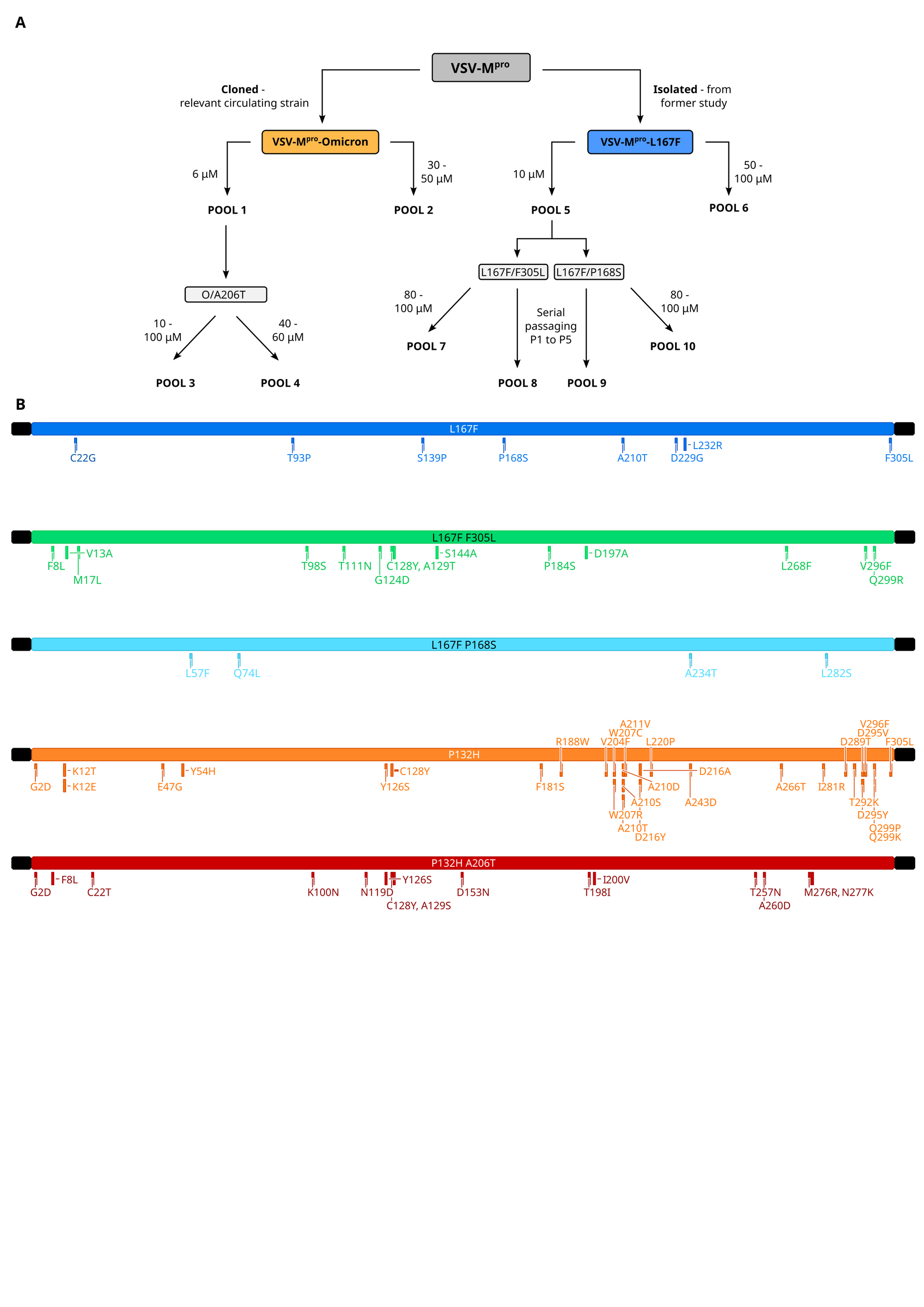

Supplement: S2 Fig — (A) Schematic representation of the selection experiments. Mutant pools are described in more detail in S1 Table. (B) Schematic representation of the different parental proteases selected and used for further selection experiments with nirmatrelvir. From top to bottom: L167F-Mpro, L167F/P168S-Mpro, L167F/F305L-Mpro, P132H-Mpro (Omicron-Mpro) and O/A206T-Mpro. Mutations are represented by column-like symbols along the proteases, according to their position in the Mpro sequence. (TIF) [file ppat.1012522.s002.tif]

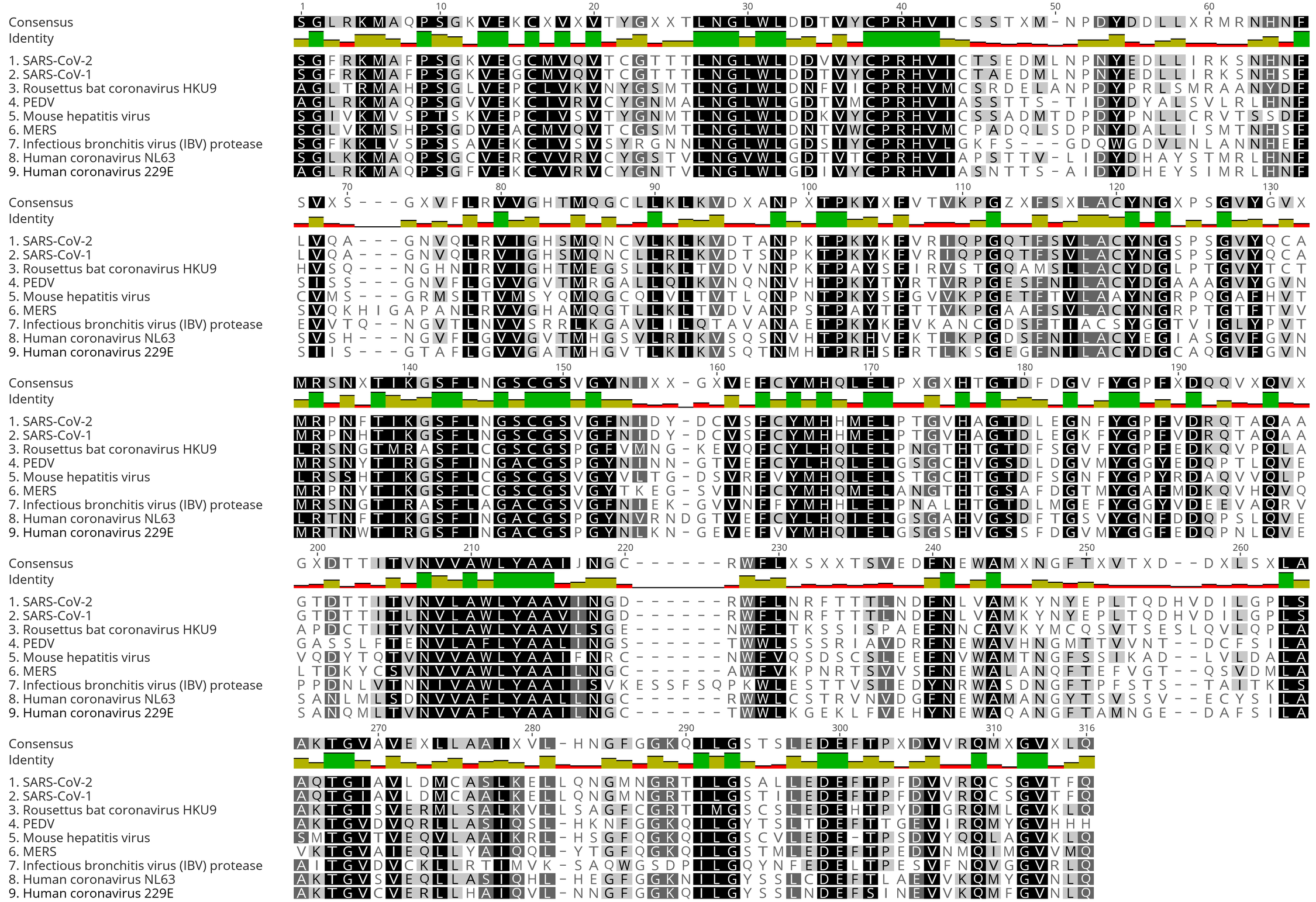

Supplement: S3 Fig — MUltiple Sequence Comparison by Log-Expectation (MUSCLE) sequence alignment of SARS-CoV-2, SARS-CoV-1, Bat-CoV HKU9, PEDV, MHV (Mouse hepatitis virus), MERS, IBV, NL63 and 229E shows areas of conservation and amino acid variability. (TIF) [file ppat.1012522.s003.tif]

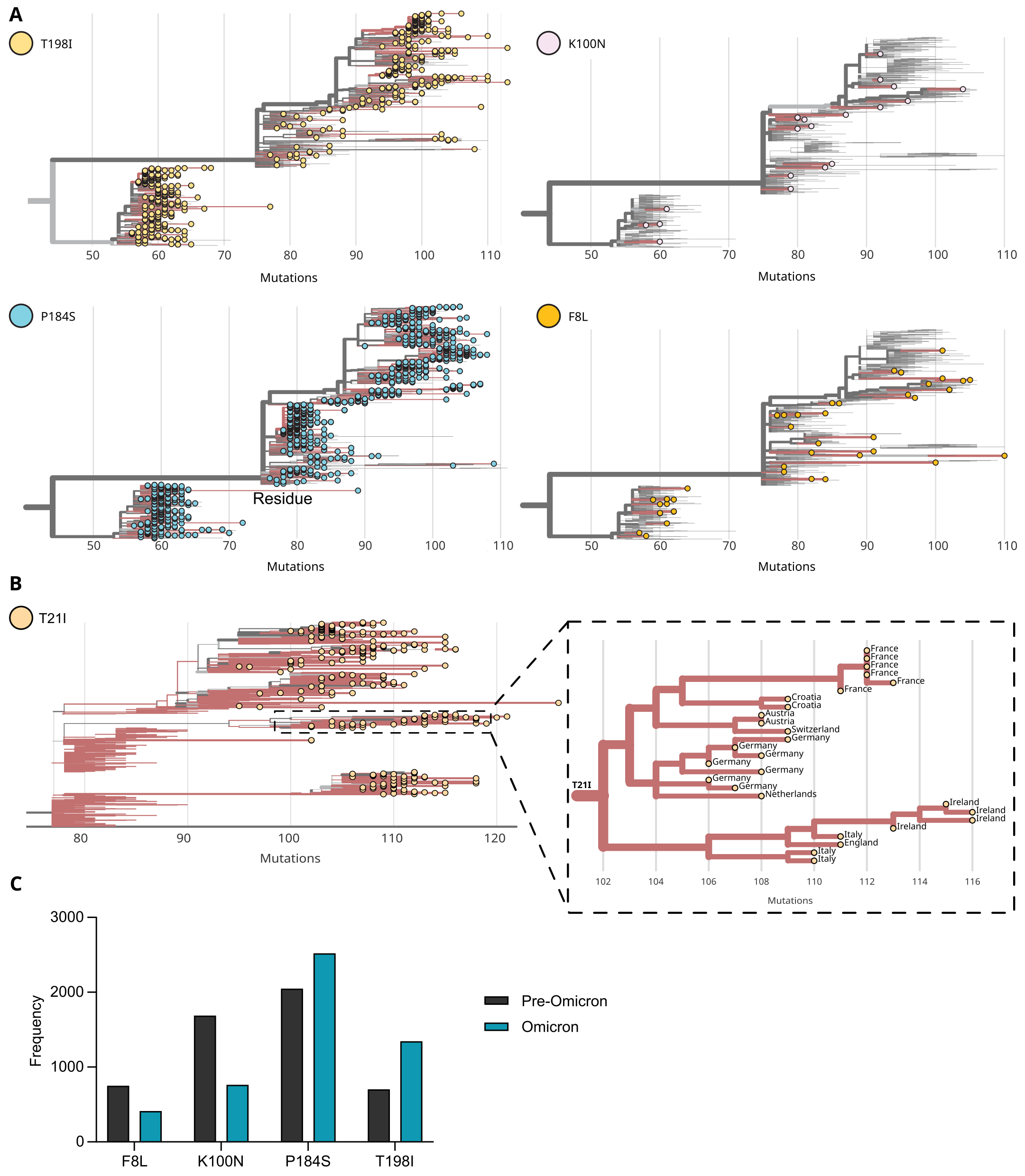

Supplement: S4 Fig — (A) Phylogenetic subtree of nsp5/Mpro-T198I, -P184S, -K100N, -F8L substitutions generated with the Ultrafast Sample placement on Existing tRee (UShER) tool (GISAID, 18th January 2023). Only sequences deposited after the Omicron emergence were used. (B) Phylogenetic subtree of nsp5/Mpro-T198I and magnified view of the T21I subtree areas, showing transmission of this variant from a single founder event. (C) Frequency ratio of T198I, P184S, K100N, and F8L mutations compared with pre- and post-Omicron variant surge (GISAID, 18th of January 2023). (TIF) [file ppat.1012522.s004.tif]

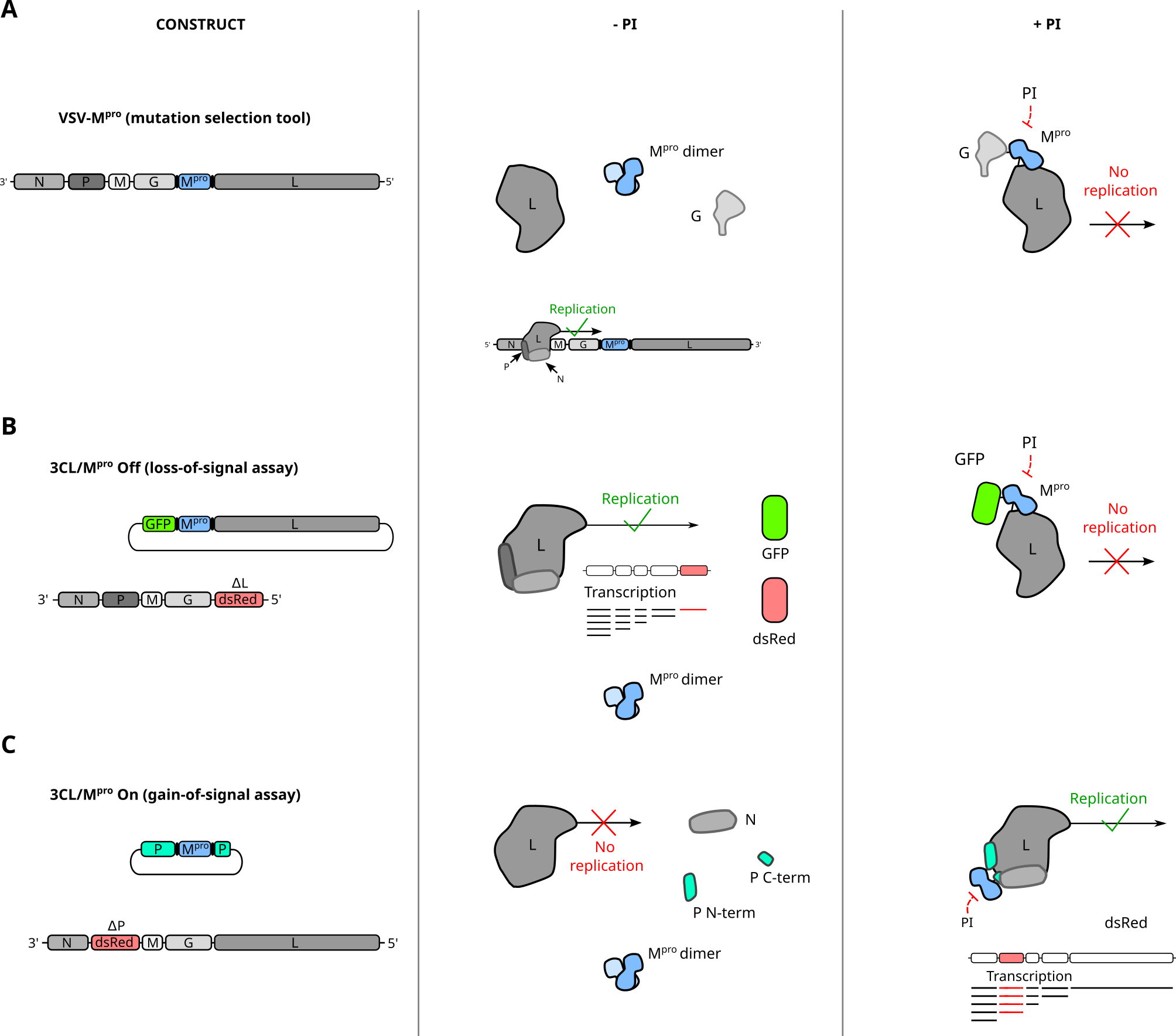

Supplement: S5 Fig — From left to right, schematic representation of: construct(s), mechanism in the absence of inhibitor, mechanism in the presence of inhibitor. (A) VSV-Mpro (mutation selection tool) construct and mechanism. Without inhibitor, Mpro processes G-Mpro-L and the virus replicates. After an inhibitor is applied, Mpro is inactive and viral replication is blocked. (B) Graphical representation of the loss-of-signal cellular assay mechanism: VSV-ΔL-dsRed + G-Mpro-L are added to cells. Without inhibitor applied, Mpro processes GFP-Mpro-L and VSV-ΔL-dsRed replicates, expressing dsRed. After an inhibitor is applied, Mpro is inactive and VSV-ΔL-dsRed replication is turned off. (C) Graphical representation of the gain-of-signal cellular assay mechanism: VSV-ΔP-dsRed + P:Mpro:P are added to cells. Without inhibitor, Mpro cleaves the phosphoprotein (P) in two pieces, impairing viral replication of replication-incompetent VSV-ΔP-dsRed. After an inhibitor is applied, Mpro is inactive, P is intact and VSV-ΔP-dsRed replication is turned on and expresses dsRed. (TIF) [file ppat.1012522.s005.tif]

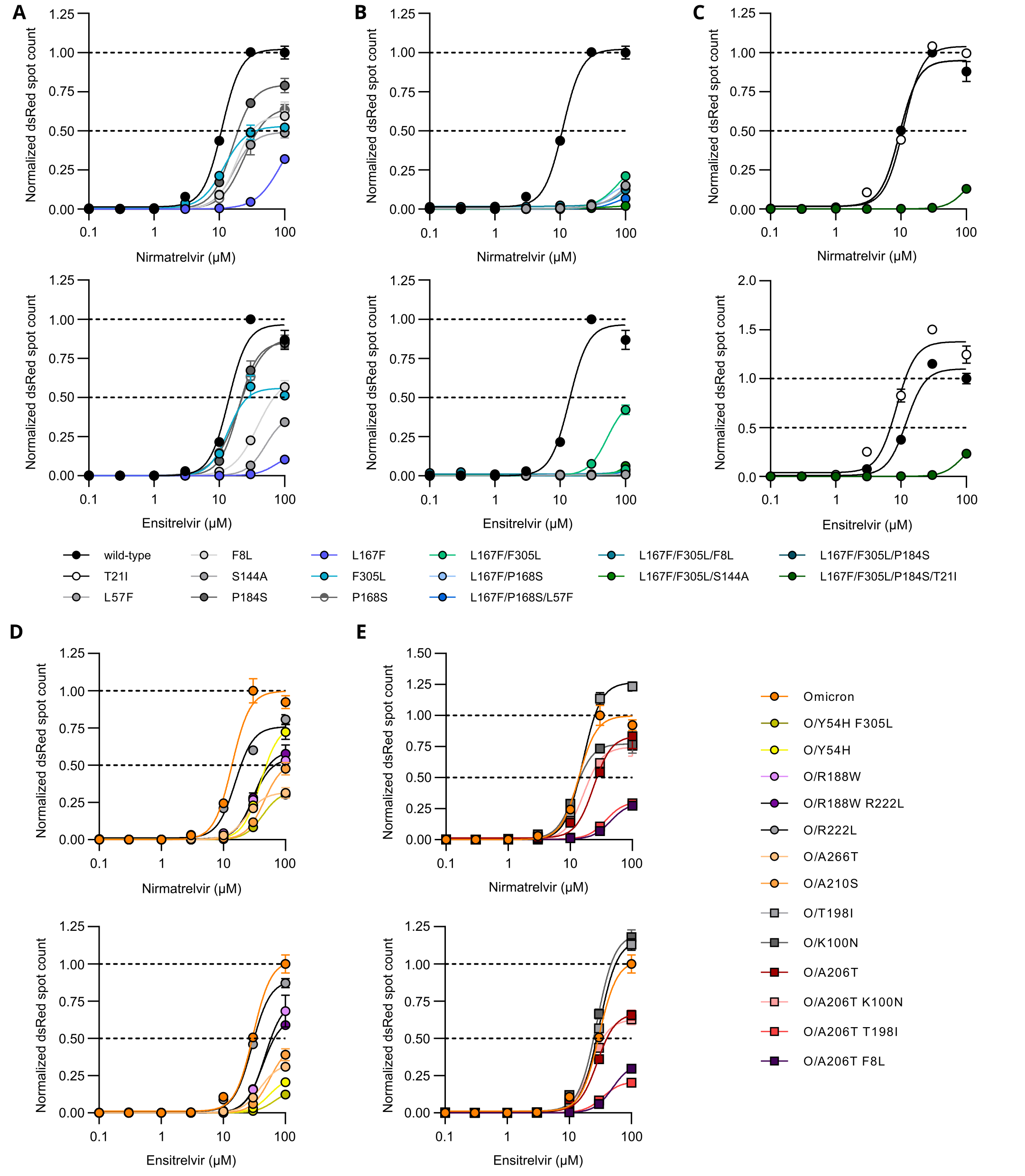

Supplement: S6 Fig — (A) Gain-of-signal assay results are shown for Mpro WT and L167F, F305L, F8L, L57F, P184S, P168S mutants against the protease inhibitors nirmatrelvir (top) and ensitrelvir (bottom). Representative experiment is shown. Data is presented as mean ± SEM of 2 biologically independent replicates per condition. (B) Gain-of-signal assay results are shown for Mpro WT and S144A, L167F/F305L, L167F/P168S, L167F/F305L/S144A, L167F/F305L/P184S, L167F/F305L/F8L, L167F/F305L/L57F mutants against the protease inhibitors nirmatrelvir (top) and ensitrelvir (bottom). Representative experiment is shown. Data is presented as mean ± SEM of 2 biologically independent replicates per condition. (C) Gain-of-signal assay results are shown for Mpro WT and T21I, L167F/F305L/P184S/T21I mutants against the protease inhibitors nirmatrelvir (top) and ensitrelvir (bottom). Representative experiment is shown. Data is presented as mean ± SEM of 2 / 3 biologically independent replicates per condition. (D) Gain-of-signal assay results are shown for Omicron-Mpro and O/A266T, O/A210S, O/Y54H, O/Y54H+F305L, O/R188W, O/R222L, O/R188W/R222L mutants against the protease inhibitor nirmatrelvir (top) and ensitrelvir (bottom). Representative experiment is shown. Data is presented as mean ± SEM of 2 biologically independent replicates per condition. (E) Gain-of-signal assay results are shown for Omicron-Mpro and O/A206T, O/T198I, O/K100N, O/A206T/F8L, O/A206T/K100N mutants against the protease inhibitor nirmatrelvir (top) and ensitrelvir (bottom). Representative experiment is shown. Data is presented as mean ± SEM of 3 biologically independent replicates per condition. (TIF) [file ppat.1012522.s006.tif]

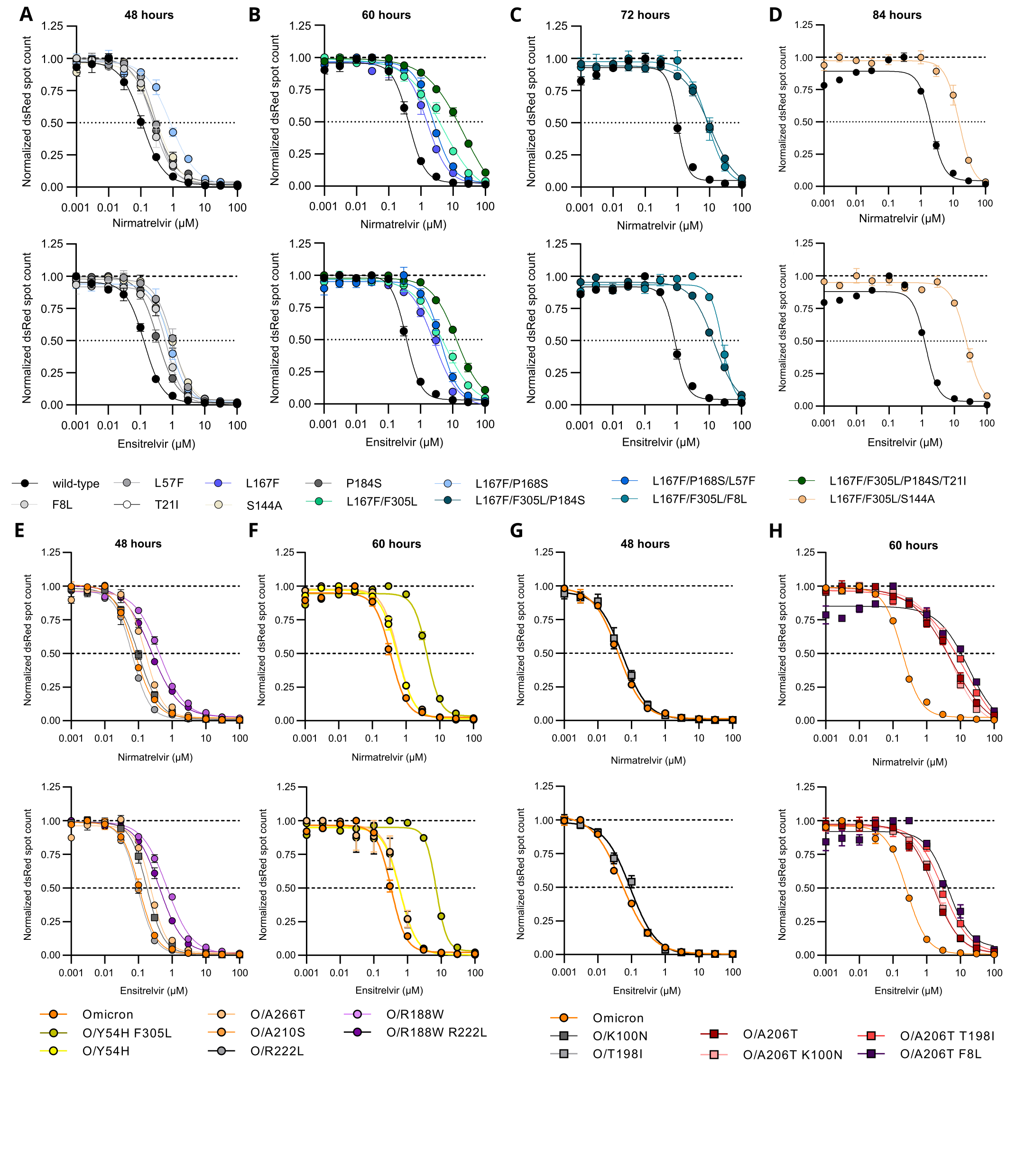

Supplement: S7 Fig — (A) Representative loss-of-signal assay results at 48 hours post infection (hpi) are shown for Mpro WT and F8L, L57F, T21I, P184S, S144A mutants against the protease inhibitors nirmatrelvir (top) and ensitrelvir (bottom). Data is presented as SEM of n = 3/n = 4 biologically independent replicates per condition. Fold-changes were calculated using the IC50 value of WT Mpro at this timepoint. (B) Representative loss-of-signal assay results at 60 hpi are shown for Mpro WT and L167F, L167F/F305L, L167F/P168S/L57F, L167F/F305L/P184S/T21I mutants against the protease inhibitors nirmatrelvir (top) and ensitrelvir (bottom). Data is presented as SEM of n = 3/n = 4 biologically independent replicates per condition. Fold-changes were calculated using the IC50 value of WT Mpro at this timepoint. (C) Representative loss-of-signal assay results at 72 hpi are shown for Mpro WT, L167F/F305L/F8L and L167F/F305L/P184S mutant against the protease inhibitors nirmatrelvir (top) and ensitrelvir (bottom). Data is presented as SEM of n = 3/n = 4 biologically independent replicates per condition. Fold-changes were calculated using the IC50 value of WT Mpro at this timepoint. (D) Representative loss-of-signal results at 84 hpi are shown for Mpro WT and L167F/F305L/S144A mutants against the protease inhibitors nirmatrelvir (top) and ensitrelvir (bottom). Data is presented as SEM of n = 3/n = 4 biologically independent replicates per condition. Fold-changes were calculated using the IC50 value of WT Mpro at this timepoint. (E) Representative loss-of-signal assay results at 48 hpi are shown for Omicron-Mpro and O/A266T, O/A210S, O/Y54H, O/K100N, O/Y54H+F305L mutants against the protease inhibitor nirmatrelvir (top) and ensitrelvir (bottom). Data is presented as SEM of n = 3/n = 4 biologically independent replicates per condition. Fold-changes were calculated using the IC50 value of Omicron Mpro at this timepoint. (F) Representative loss-of-signal assay results at 48 hpi are shown for Omicron-Mpr [file ppat.1012522.s007.tif]

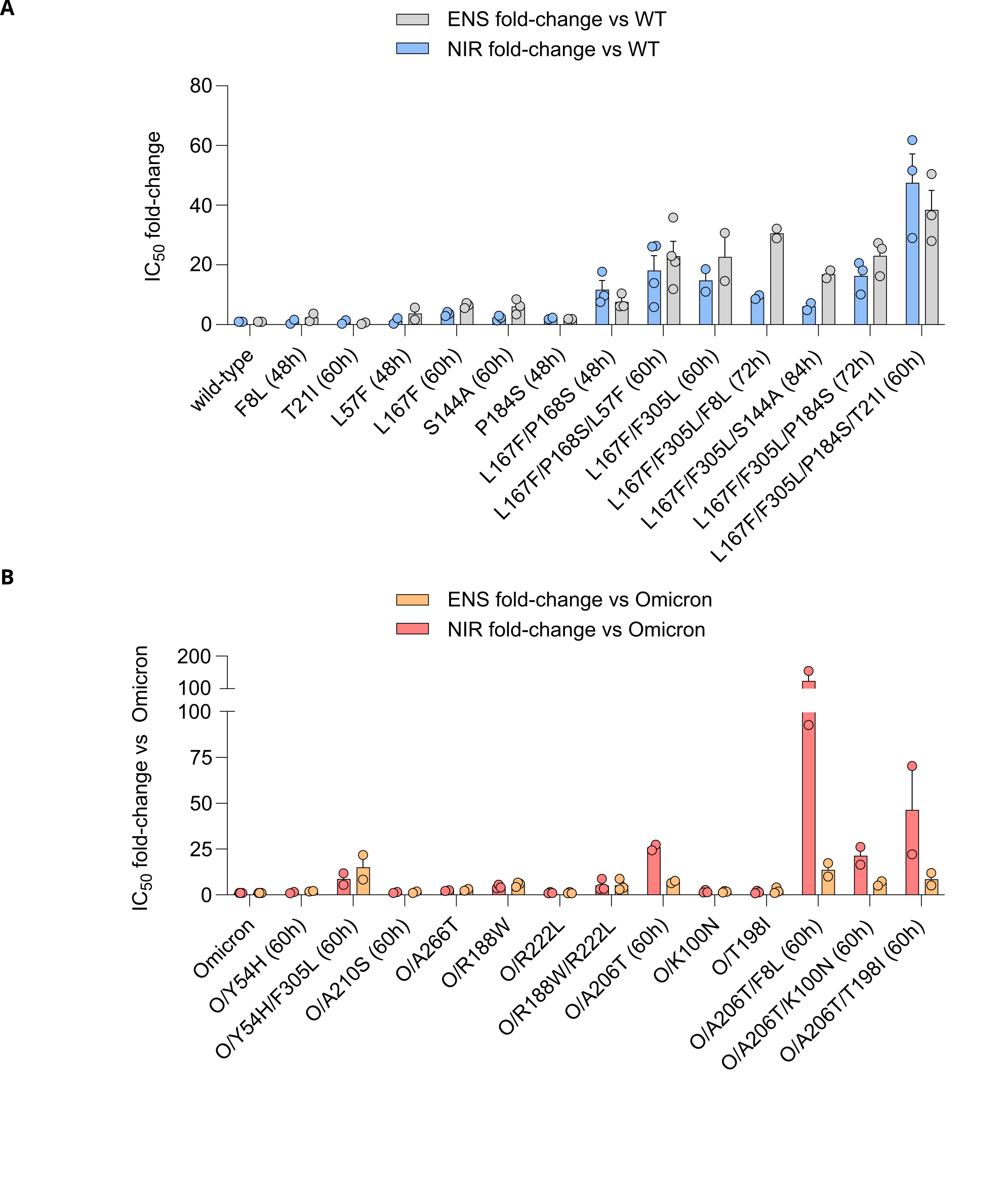

Supplement: S8 Fig — (A) Loss-of-signal assay results are shown for all the WT Mpro mutants. Data is presented as mean and SD of n = 2 / n = 3 or n = 4 independent experiments. (B) Loss-of-signal assay results are shown for all the Omicron Mpro mutants. Data is presented as mean and SD of n = 2 / n = 3 independent experiments. (TIF) [file ppat.1012522.s008.tif]

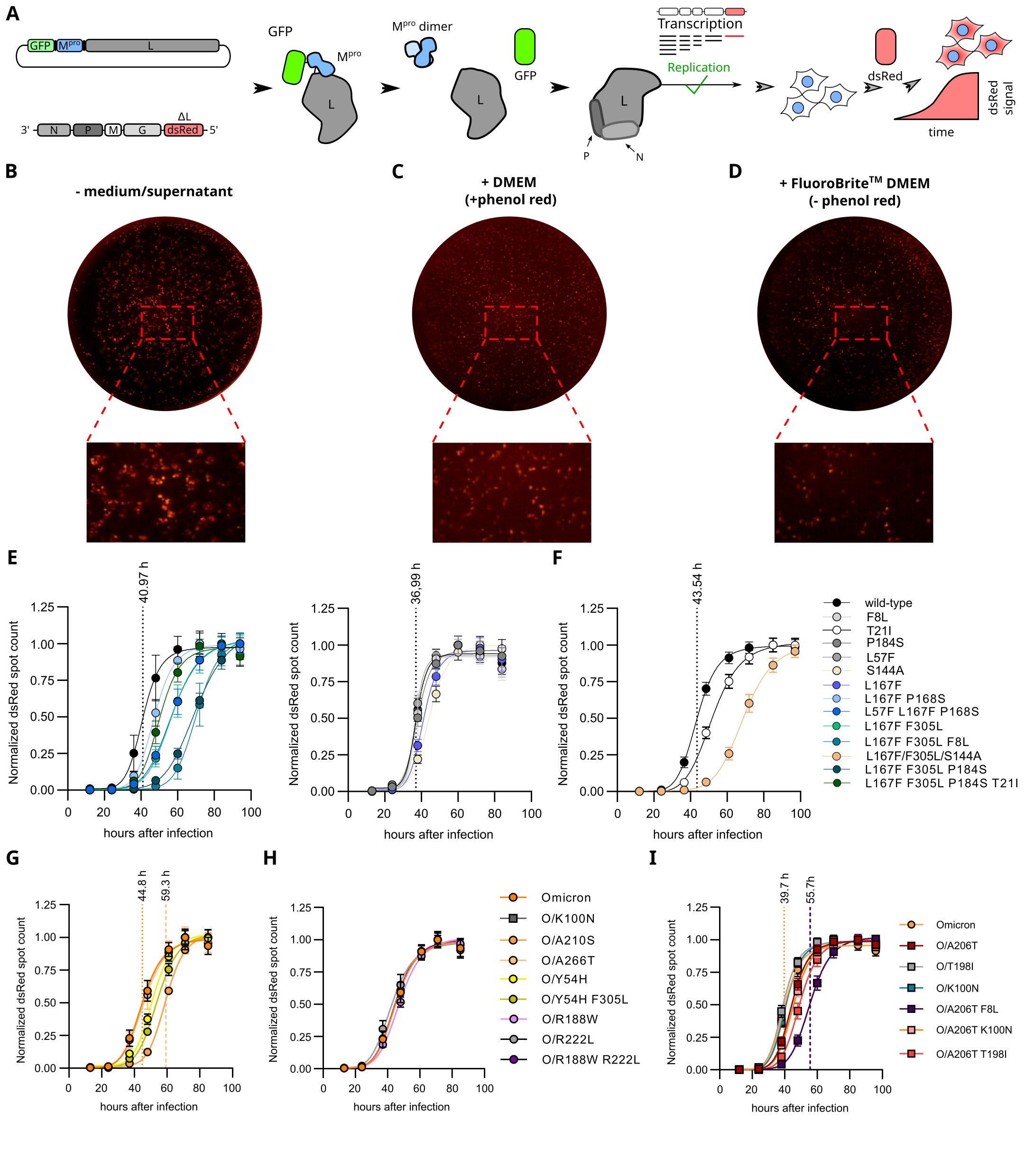

Supplement: S9 Fig — (A) Schematic representation of the Mpro-Off assay adaptation to a viral replication kinetic measurement based on Mpro activity. Mpro-Off transfected cells are infected with VSV-ΔL-dsRed and the signal is read out over time. No protease inhibitor is applied. (B) Representative photo, and magnified view (below), taken with the ELISpot reader (FluoroSpot X suite) of a well after removing the supernatant. (C) Representative photo, and magnified view (below), taken with the ELISpot reader (FluoroSpot X suite) of a well where the supernatant was not removed (DMEM, + phenol red). (D) Representative photo, and magnified view (below), taken with the ELISpot reader (FluoroSpot X suite) of a well where the supernatant was not removed (FluoroBrite DMEM,—phenol red). (E) Representative replication kinetics fitting curves of WT Mpro and mutants F8L, T21I, P184S, L57F, S144A, L167F, L167F/P168S, L167F/P168S/L57F, L167F/F305L, L167F/F305L/F8L, L167F/F305L/S144A, L167F/F305L/P184S, L167F/F305L/P184S/T21I (± SD; n = 12 biologically independent replicates). The dotted line represents the TM50 value related to the WT Mpro. (G) Replication kinetics fitting curves of Omicron, O/Y54H, O/Y54H/F305L, O/A210S, O/A266T, O/K100N Mpro mutants (± SD; n = 8 biologically independent replicates). The dotted lines represent the TM50 value related to the Omicron main protease and the mutant O/A210S. (H) Replication kinetics fitting curve of Omicron, O/R188W, O/R222L, O/R188W/R222L Mpro mutants (± SEM; n = 8 biologically independent replicates). (I) Replication kinetics fitting curves of Omicron, O/A206T, O/A206T/F8L, O/A206T/T198I, O/A206T/K100N, O/T198I Mpro mutants (± SEM; n = 8 biologically independent replicates). The dotted lines represent the TM50 value related to the Omicron main protease and the mutant O/A206T/F8L. (TIF) [file ppat.1012522.s009.tif]

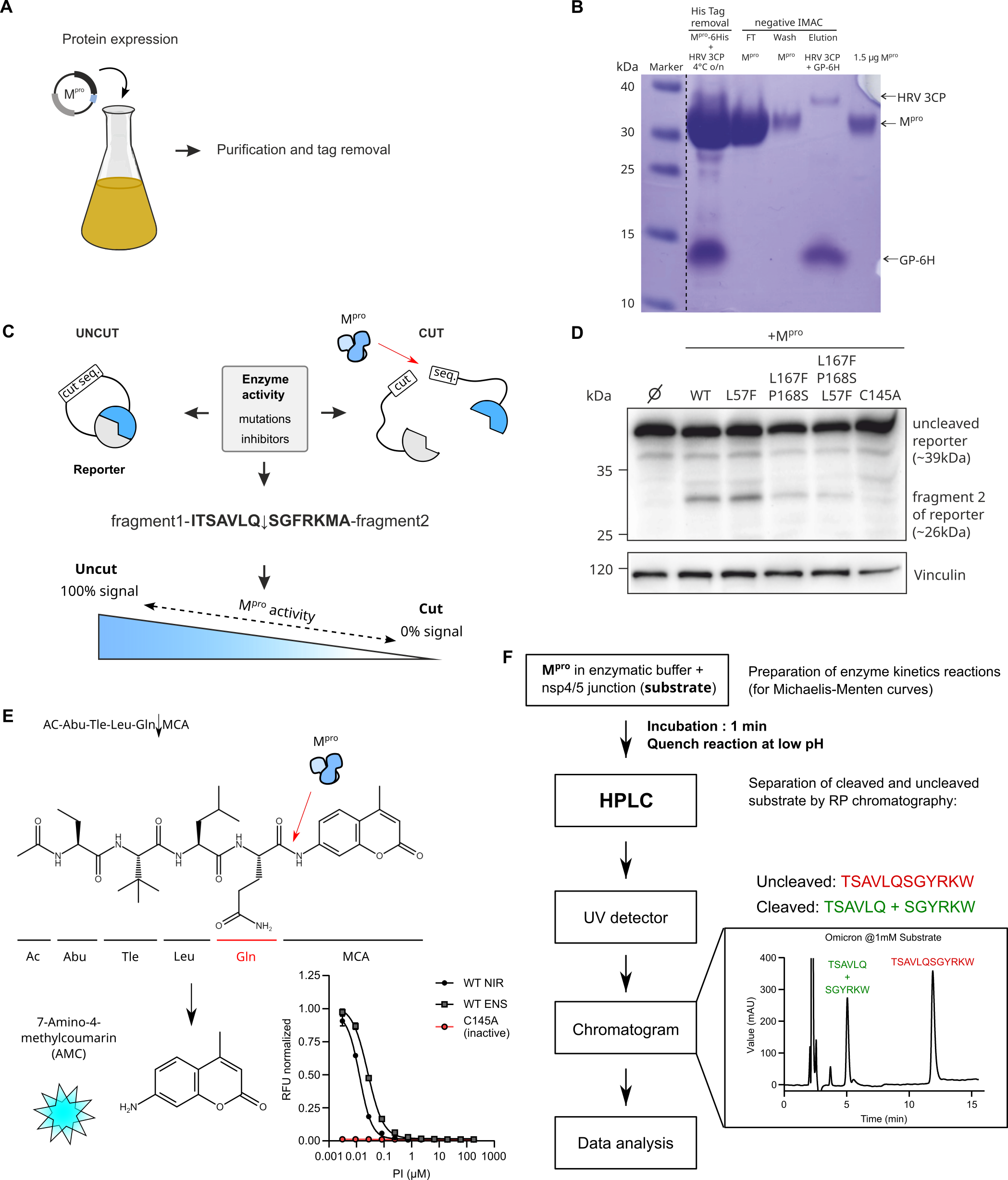

Supplement: S10 Fig — (A) Following recombinant protein expression, Mpro variants were purified using a FPLC system for subsequent assays employing the recombinant protease. (B) Coomassie stained denaturing protein gel detailing purification steps of Mpro. (C) Scheme of the Mpro-cutting reporter. The cutting sequence of Mpro is flanked by two fragments of the reporter protein. Incubation of the reporter with purified Mpro leads to reporter cleavage. Protease inhibitor binding and mutations can stop the cleavage event. (D) Proteolytic activity assessment via western blot of uncleaved reporter protein compared to WT Wuhan-1 Mpro and mutants thereof. (E) Fluorogenic substrate peptide coupled with MCA, which releases fluorescent AMC upon cleavage by Mpro. Dose response experiments of WT and C145A mutant (catalytically inactive) were performed with nirmatrelvir and ensitrelvir (± SEM; n = 2 technical replicates). (F) Schematic representation of the HPLC-based peptide cleavage assay. Magnified, a representative chromatogram showing the cleaved peptide (green text) and the uncleaved peptide (red text) peaks. (TIF) [file ppat.1012522.s010.tif]

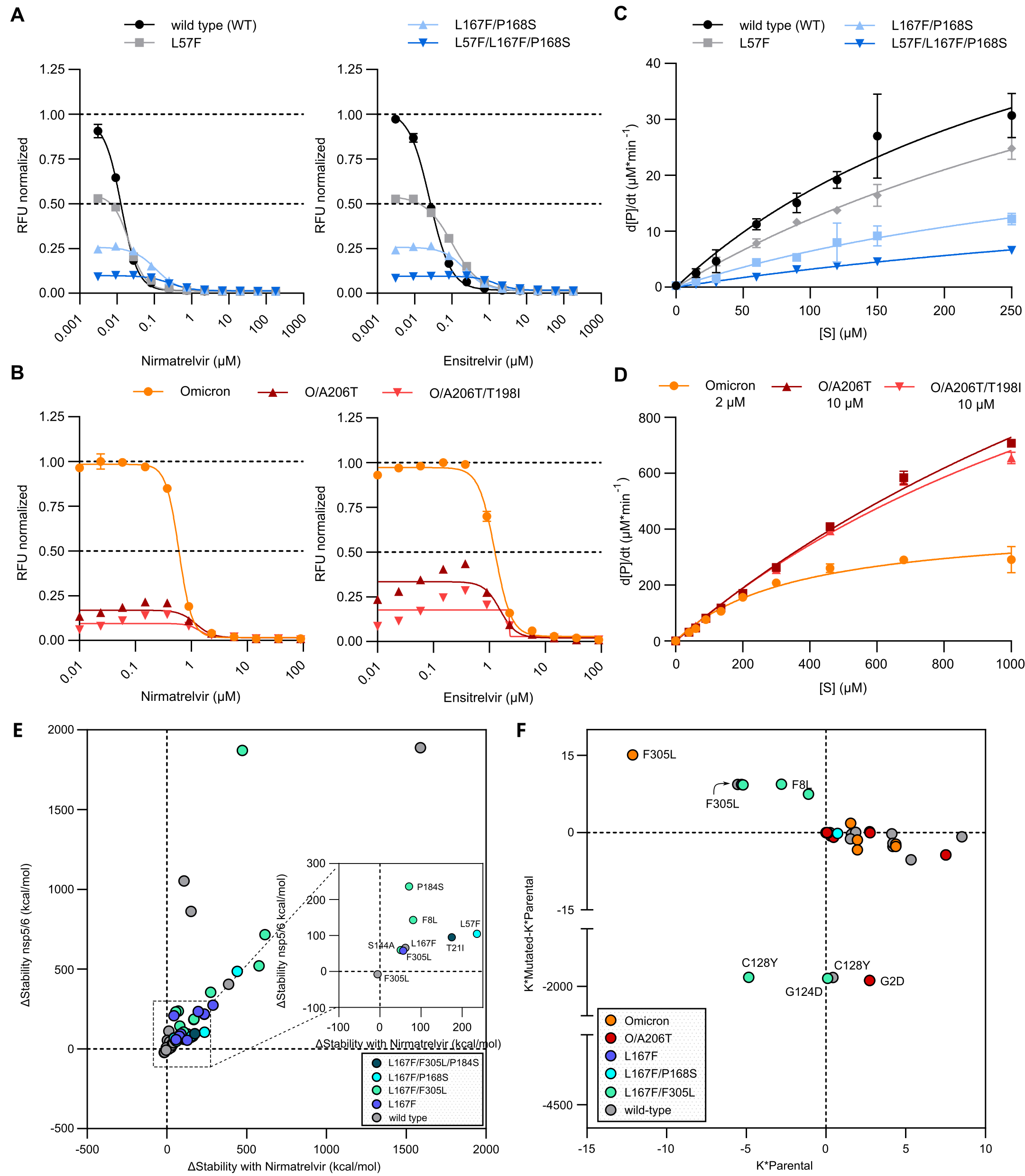

Supplement: S11 Fig — (A) Left: dose response experiment of WT, L57F, L167F/P168S and L167F/P168S/L57F with nirmatrelvir (± SD; n = 2 technical replicates). Right: dose response experiment of WT, L57F, L167F/P168S and L167F/P168S/L57F with ensitrelvir (± SD; n = 2 technical replicates). (B) Left: dose response experiment of Omicron, O/A206T and O/A206T/T198I with nirmatrelvir (± SD; n = 2 technical replicates). Right: dose response experiment of Omicron, O/A206T and O/A206T/T198I with nirmatrelvir (± SD; n = 2 technical replicates). (C) Michaelis-Menten curves of WT, L57F, L167F/P168S and L167F/P168S/L57F proteases (± SD, n = 3 independent experiments). All the proteases were tested at 2 μM. (D) Michaelis-Menten curves of Omicron, A206T, A206T/T198I proteases (± SD, n = 3 independent experiments). Omicron protease was tested at 2 μM, whereas O/A206T and O/A206T/T198I were tested at 10 μM. (E) Plot of Δ_Stability values for mutations introduced to the nsp5/6-Mpro structure (PDB entry 7DVW) and in the nirmatrelvir bound structure (PDB entry 8DZ2). Inside the plot, a magnified subplot highlighting some data points. Some of the mutations that were investigated in this work are labelled here. (F) Dimerization affinity plot: positive values mean increase in dimerization affinity, negative values indicate decreased or impaired affinity. Each data point represents a mutation, which is colored based on the parental protease they have arose from (i.e. F8L is colored in light sea green and it represent the triple mutant L167F/F305L/F8L). (TIF) [file ppat.1012522.s011.tif]

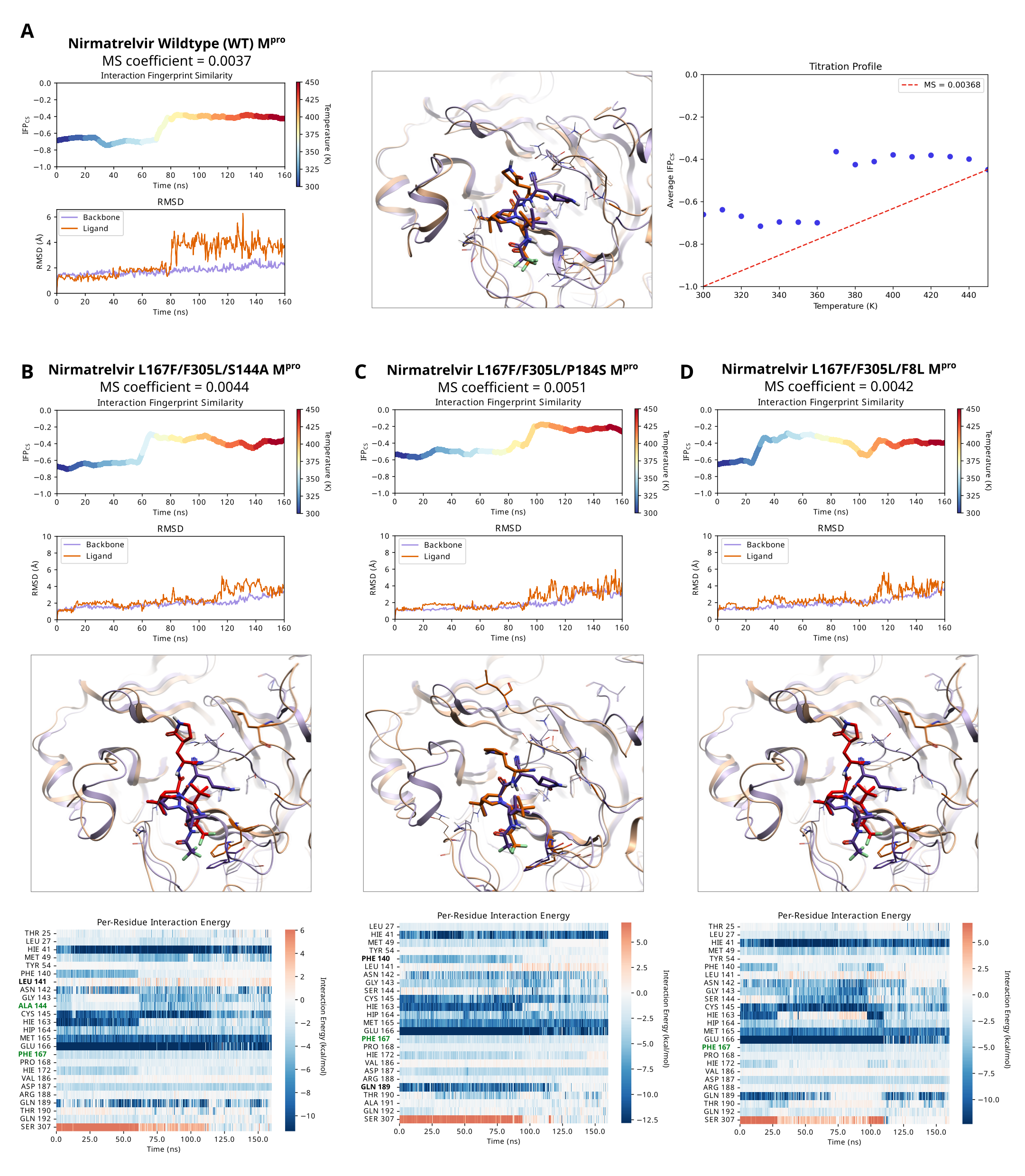

Supplement: S12 Fig — (A) Simulation with the WT protease. Left: rainbow plot and Root Mean Square Deviation (RMSD) of backbone and ligand. Middle: structural overlay of pre- and post-TTMD simulation. Right: titration profile, from which the MS coefficient can be extrapolated. (B) Simulation with the L167F/F305L/S144A mutant protease. Top: rainbow plot and Root Mean Square Deviation (RMSD) of backbone and ligand. Middle: structural overlay of pre- and post-TTMD simulation. Bottom: heat map with interaction energies between the ligand and the surrounding residues. (C) Simulation with the L167F/F305L/P184S mutant protease. Top: rainbow plot and Root Mean Square Deviation (RMSD) of backbone and ligand. Middle: structural overlay of pre- and post-TTMD simulation. Bottom: heat map with interaction energies between the ligand and the surrounding residues. (D) Simulation with the L167F/F305L/F8L mutant protease. Top: rainbow plot and Root Mean Square Deviation (RMSD) of backbone and ligand. Middle: structural overlay of pre- and post-TTMD simulation. Bottom: heat map with interaction energies between the ligand and the surrounding residues. (TIF) [file ppat.1012522.s012.tif]

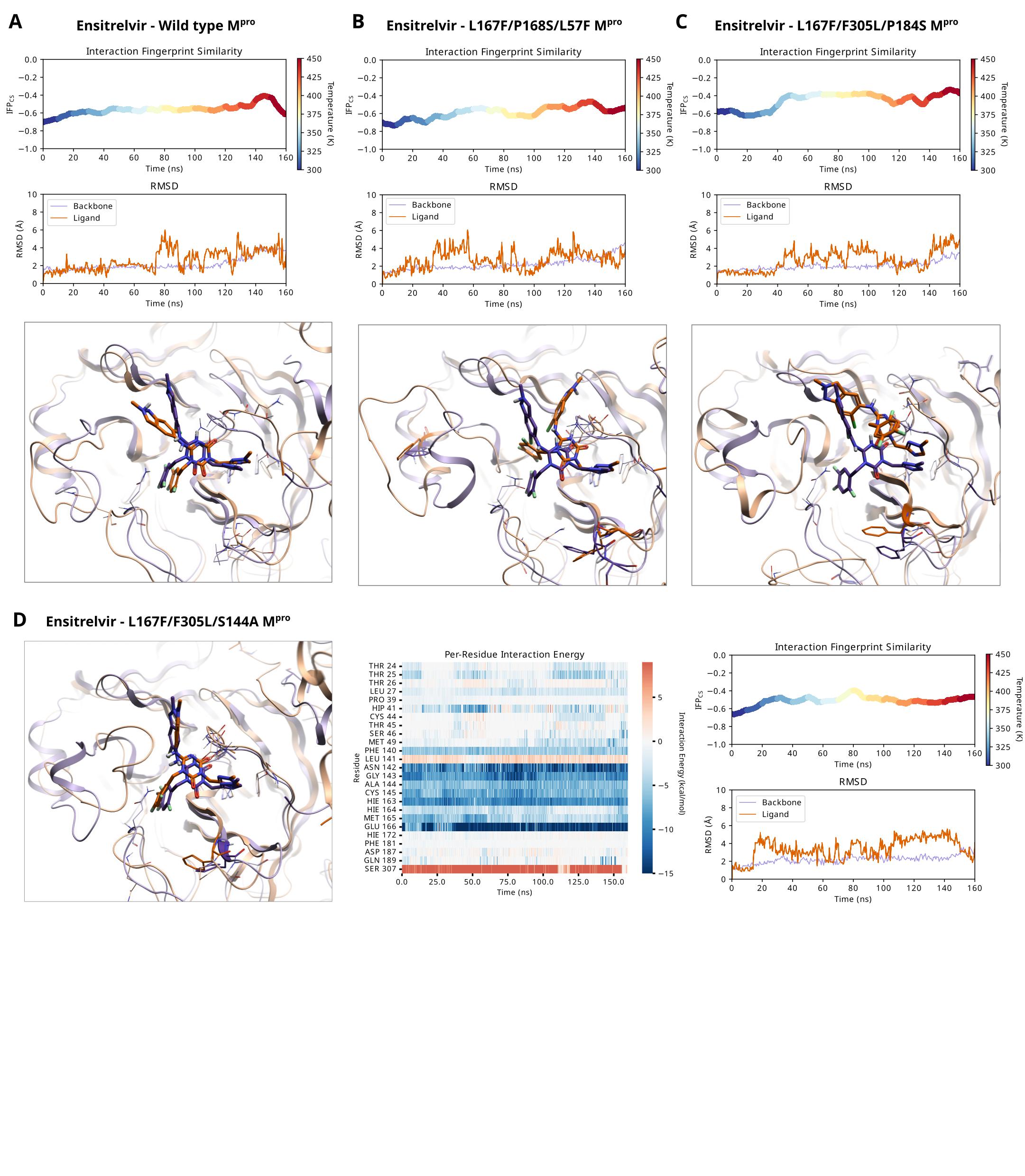

Supplement: S13 Fig — TTMD simulation data of the ensitrelvir-WT-Mpro (A), ensitrelvir- L167F/P168S/L57F -Mpro (B), ensitrelvir-L167F/F305L/P184S-Mpro (C) ensitrelvir-L167F/F305L/P184S-Mpro (D) complexes. (TIF) [file ppat.1012522.s013.tif]
