# Supplementary material for: A comprehensive study of SARS-CoV-2 main protease (Mpro) inhibitor-resistant mutants selected in a VSV-based system
Source: PLoS Pathog. 2024 Sep 11;20(9):e1012522. doi: 10.1371/journal.ppat.1012522 (PMC11407635; doi:10.1371/journal.ppat.1012522)
Supplement: S1 Table — (DOCX) [file ppat.1012522.s014.docx]

| **Parental virus** | **POOL** | **[NIR] μM** | **Sample mutation 1** | **Sample mutation 2** | **Sample mutation 3** |
| --- | --- | --- | --- | --- | --- |
| **VSV-M^pro^-Omicron** | **1** | **6** | K12T | F305L |  |
|  |  |  | E47G | C300R |  |
|  |  |  | Y126S |  |  |
|  |  |  | C128Y |  |  |
|  |  |  | V204F |  |  |
|  |  |  | A206T |  |  |
|  |  |  | W207C |  |  |
|  |  |  | A210T |  |  |
|  |  |  | A211V |  |  |
|  |  |  | L220P |  |  |
|  |  |  | I281R |  |  |
|  |  |  | D295Y |  |  |
|  |  |  | V296F |  |  |
|  |  |  | Q299P |  |  |
|  |  |  | Q299K |  |  |
|  | **2** | **30** | K12E | I281R | D295G |
|  |  |  | Y54H | A210T |  |
|  |  |  | Y54H |  |  |
|  |  |  | Y54H | A210T |  |
|  |  |  | Y54H | R188W |  |
|  |  |  | Y54H | A206T |  |
|  |  |  | Y54H | A206T |  |
|  |  |  | Y54H | A234D |  |
|  |  |  | C128Y |  |  |
|  |  |  | V204F | D289T |  |
|  |  |  | W207R |  |  |
|  |  |  | D216Y |  |  |
|  |  |  | L220P |  |  |
|  |  |  | D289T |  |  |
|  |  |  | D295V |  |  |
|  |  | **40** | G2D | Y54H |  |
|  |  |  | Y54H | Q299R |  |
|  |  |  | Y54H | A210T |  |
|  |  |  | A206T | A266T |  |
|  |  |  | A210D |  |  |
|  |  | **50** | F181S |  |  |
|  |  |  | A206T |  |  |
|  |  |  | A210S |  |  |
|  |  |  | D216A | T292K |  |
| **Parental virus** | **POOL** | **[NIR] μM** | **Sample mutation 1** | **Sample mutation 2** | **Sample mutation 3** |
| **VSV-M^pro^-O/A206T** | **3** | **10 − 20** | G2D |  |  |
|  |  |  | F8L |  |  |
|  |  |  | N119D |  |  |
|  |  |  | A129S |  |  |
|  |  |  | I200V |  |  |
|  |  |  | M276R |  |  |
|  | **4** | **40** | C22T |  |  |
|  |  |  | N119D |  |  |
|  |  |  | A260D |  |  |
|  |  |  | A260D |  |  |
|  |  | **50** | C128Y |  |  |
|  |  |  | D153N |  |  |
|  |  |  | T198I |  |  |
|  |  | **60** | F3S |  |  |
|  |  |  | K100N |  |  |
|  |  |  | N119D |  |  |
|  |  |  | Y126S |  |  |
|  |  |  | T257N |  |  |
|  |  |  | N277K |  |  |
| **Parental virus** | **POOL** | **[NIR] μM** | **Sample mutation 1** | **Sample mutation 2** | **Sample mutation 3** |
| **VSV-M^pro^-L167F** | **5** | **10** | P168S |  |  |
|  |  |  | F305L |  |  |
|  | **6** | **50** | C22G |  |  |
|  |  |  | S139P |  |  |
|  |  |  | A210T |  |  |
|  |  | **80** | L232R |  |  |
|  |  | **90** | T93P |  |  |
|  |  | **100** | A210T |  |  |
|  |  |  | D229G |  |  |
| **Parental virus** | **POOL** | **[NIR] μM** | **Sample mutation 1** | **Sample mutation 2** | **Sample mutation 3** |
| **VSV-M^pro^-L167F/P168S** | **7** | **90** | L57F |  |  |
|  |  |  | A234T |  |  |
|  | **8** | **P1 - P4 (conc.: 40, 50, 60, 60)** | Q74L |  |  |
|  |  |  | L282S |  |  |
| **Parental virus** | **POOL** | **[NIR] μM** | **Sample mutation 1** | **Sample mutation 2** | **Sample mutation 3** |
| **VSV-M^pro^-L167F/F305L** | **9** | **P1 - P4 (conc.: 60, 60, 70, 70)** | F8L |  |  |
|  |  |  | M17L | P184S |  |
|  |  |  | T98S |  |  |
|  |  |  | T111N |  |  |
|  |  |  | G124D |  |  |
|  |  |  | C128Y |  |  |
|  |  |  | A129T | Q299R |  |
|  |  |  | S144A |  |  |
|  |  |  | D197A |  |  |
|  |  |  | L268F |  |  |
|  |  |  | V296F |  |  |
|  | **10** | **80** | L268F |  |  |
|  |  | **90** | V13A |  |  |

**S1 Table**: VSV-M^pro^ mutants generated during selection experiments with the indication of at which passage and concentration they have been selected.
