# Supplementary material for: A comprehensive study of SARS-CoV-2 main protease (Mpro) inhibitor-resistant mutants selected in a VSV-based system
Source: PLoS Pathog. 2024 Sep 11;20(9):e1012522. doi: 10.1371/journal.ppat.1012522 (PMC11407635; doi:10.1371/journal.ppat.1012522)
Supplement: S2 Table — (DOCX) [file ppat.1012522.s015.docx]

| Mutation | Variant of concern | Count GISAID 16.05.2024 |
| --- | --- | --- |
| T21I | Alpha | 1103 |
|  | Delta | 3484 |
|  | Gamma | 979 |
|  | Omicron | 7424 |
| F8L | Delta | 701 |
|  | Omicron | 91 |
| K100N | Delta | 2253 |
|  | Omicron | 79 |
| P168S | Delta | 272 |
|  | Omicron | 161 |
| A194S | Delta | 227 |
|  | Omicron | 273 |
| P184S | Alpha | 433 |
|  | Delta | 1869 |
|  | Omicron | 2042 |
| T198I | Delta | 836 |
|  | Omicron | 1256 |
| A210S | Alpha | 74 |
|  | Delta | 110 |
|  | Omicron | 497 |
| R222L | Delta | 211 |
|  | Omicron | 486 |
| A234T | Delta | 414 |
|  | Omicron | 473 |
| A266T | Delta | 65 |
|  | Omicron | 40 |

**S2 Table**. Break-down of mutations with counts >500 in GISAID according to their appearance in a variant of concern.
