# Supplementary material for: A comprehensive study of SARS-CoV-2 main protease (Mpro) inhibitor-resistant mutants selected in a VSV-based system
Source: PLoS Pathog. 2024 Sep 11;20(9):e1012522. doi: 10.1371/journal.ppat.1012522 (PMC11407635; doi:10.1371/journal.ppat.1012522)
Supplement: S3 Table — (DOCX) [file ppat.1012522.s016.docx]

| Name | Sequence (5’-3’ direction) |
| --- | --- |
| 33n-before-KpnI-for | GAACCGGTCCTGCTTTCACC |
| G-cut1-rev | CATTTTTCTAAAACCACTCTGCAAAACAGCTGAGGTGATCTTTCCAAGTCGGTTC |
| cut1-for | ATCACCTCAGCTGTTTTGCAG |
| cut2-L-rev | GTCGGTCTCAAAATCGTGGACTTCCATGATTGTTCTTTTCACTGCACTTTG |
| cut2-L-for | AGTGCAGTGAAAAGAACAATCATGGAAGTCCACGATTTTGAG |
| 33n-after-HpaI-rev | GATGTTGGGATGGGATTGGC |
| Omicron-for | CAATGTGCTATGAGGCACAATTTCAC |
| Omicron-rev | CTTAATAGTGAAATTGTGCCTCATAGC |
| blasticidin-for | CATTCGATTAGTGAACGGATCTC |
| L rev | GATGTTGGGATGGGATTGGC |
| hygro-P-for | CTGTTTTGACCTCCATAGAAGATTCTAGAGCTAGCATGGATAATCTCACAAAAGTTC |
| P-hygro-rev | GAGGGAGAGGGGCGGATCCCCTTAATTAACTACAGAGAATATTTGACTCTCGC |
| 3CL^pro^-L167F-for | GCACCATATGGAATTTCCAACTG |
| 3CL^pro^-L167F-rev | CATGAACTCCAGTTGGAAATTCC |
| A206T for | CTATTACAGTTAATGTTTTAACTTGGTTGTACGCT |
| A206T rev | CATTTATAACAGCAGCGTACAACCAAGTTAAAACATT |
| R188W for | GGACCTTTTGTTGACTGGCAAAC |
| R188W rev | CTTGTGCTGTTTGCCAGTCAAC |
| R222L for | GGTGGTTTCTCAATCTATTTACCACAAC |
| R222L rev | GTCATTAAGAGTTGTGGTAAATAGATTGAG |
| Y54H for | CATGCTTAACCCTAATCATGAAGATTTACTC |
| Y54H rev | GACTTACGAATGAGTAAATCTTCATGATTAGGG |
| K100N for | GCCAATCCTAAGACACCTAATTATAAG |
| K100N rev | GCGAACAAACTTATAATTAGGTGTC |
| T198I for | CAGCTGGTACGGACATAAC |
| T198I rev | CATTAACTGTAATAGTTATGTCCGTACC |
| A210S for | GCTTGGTTGTACTCTGCTG |
| A210S rev | CCATTTATAACAGCAGAGTACAACC |
| A266T for | GCCGTTTTAGATATGTGTACTTCATTAAAAG |
| A266T rev | GCAGTAATTCTTTTAATGAAGTACACATATC |
| L167F, P168S for | GCACCATATGGAATTCTCAACTG |
| L167F, P168S rev | CATGAACTCCAGTTGAGAATTCC |
| F8L for | GTTTTAGAAAAATGGCATTACCATCTGG |
| F8L rev | CTCAACTTTACCAGATGGTAATGCC |
| L57F for | CCCTAATTATGAAGATTTCCTCATTCG |
| L57F rev | GATTAGACTTACGAATGAGGAAATCTTC |
| P184S for | GAAGGTAACTTTTATGGATCTTTTGTTG |
| P184S rev | GCCTGTCAACAAAAGATCCATAAAAG |
| S144A for | CATTCCTTAATGGTGCATGTGG |
| S144A rev | CACTACCACATGCACCATTAAG |
| P168S for | CACCATATGGAATTATCAACTGGAG |
| P168S rev | GCATGAACTCCAGTTGATAATTCC |
| T21I for | GGGTTGTATGGTACAAGTAATTTGTGGTAC |
| T21I rev | CGTTAAGTGTAGTTGTACCACAAATTACTTG |
| C145A for | GTTCATTCCTTAATGGTTCAGCTGGTAGTG |
| C145A rev | CTATGTTAAAACCAACACTACCAGCTGAACC |

**S3 Table**: cloning primers.
