# Supplementary material for: A comprehensive study of SARS-CoV-2 main protease (Mpro) inhibitor-resistant mutants selected in a VSV-based system
Source: PLoS Pathog. 2024 Sep 11;20(9):e1012522. doi: 10.1371/journal.ppat.1012522 (PMC11407635; doi:10.1371/journal.ppat.1012522)
Supplement: S4 Table — Table displaying all the retrieved mutations from different VSV-Mpro variants used: VSV-O/A206T-Mpro, VSV-Omicron-Mpro, VSV-L167F-Mpro, VSV-L167F/P168S-Mpro and VSV-L167F/F305L-Mpro. For each substitution, the coverage (in percentage) is reported on its right. (DOCX) [file ppat.1012522.s017.docx]

| Residue number |  |  | | VSV-M^pro^ variant (Nanopore sequencing) | | | | | | | | |
| --- | --- | --- | --- | --- | --- | --- | --- | --- | --- | --- | --- | --- |
|  | VSV-Omicron | | | | VSV-O-A206T | | VSV-L167F | | VSV-L167F/P168S | | VSV-L167F/F305L | |
| -4 |  | |  | |  |  |  |  |  |  |  |  |
| 2 | G2D | | 69.29% | | G2D | 11.89% |  |  |  |  |  |  |
| 3 |  | |  | | F3S | 22.22% |  |  |  |  |  |  |
| 8 |  | |  | | F8L | 16.76% |  |  |  |  | F8L | 8.60% |
| 12 | K12E | | 11.88% | |  |  |  |  |  |  |  |  |
| 13 |  | |  | |  |  |  |  |  |  | V13A | 11.66% |
| 17 |  | |  | |  |  |  |  |  |  | M17L | 5.13% |
| 22 |  | |  | | C22T | 21.41% | C22G | 37.40% |  |  |  |  |
| 39 |  | |  | |  |  |  |  |  |  |  |  |
| 54 | Y54H | | 28.28% | |  |  |  |  |  |  |  |  |
| 54 | Y54H | | 25.67% | |  |  |  |  |  |  |  |  |
| 54 | Y54H | | 12.00% | |  |  |  |  |  |  |  |  |
| 54 | Y54H | | 6.80% | |  |  |  |  |  |  |  |  |
| 54 | Y54H | | 6.66% | |  |  |  |  |  |  |  |  |
| 54 | Y54H | | 4.53% | |  |  |  |  |  |  |  |  |
| 54 | Y54H | | 15.51% | |  |  |  |  |  |  |  |  |
| 54 | Y54H | | 50.83% | |  |  |  |  |  |  |  |  |
| 54 | Y54H | | 12.64% | |  |  |  |  |  |  |  |  |
| 54 | Y54H | | 13.73% | |  |  |  |  |  |  |  |  |
| 57 |  | |  | |  |  |  |  | L57F | 82.27% |  |  |
| 74 |  | |  | |  |  |  |  | Q74L | 7.68% |  |  |
| 93 |  | |  | |  |  | T93P | 25.39% |  |  |  |  |
| 98 |  | |  | |  |  |  |  |  |  | T98S | 4.70% |
| 100 |  | |  | | K100N | 9.11% |  |  |  |  |  |  |
| 100 |  | |  | |  |  |  |  |  |  |  |  |
| 111 |  | |  | |  |  |  |  |  |  | T111N | 6.71% |
| 119 |  | |  | | N119D | 11.44% |  |  |  |  |  |  |
| 119 |  | |  | | N119D | 6.01% |  |  |  |  |  |  |
| 119 |  | |  | | N119D | 7.40% |  |  |  |  |  |  |
| 123 |  | |  | |  |  |  |  |  |  |  |  |
| 124 |  | |  | |  |  |  |  |  |  | G124D | 6.84% |
| 126 |  | |  | | Y126S | 12.01% |  |  |  |  |  |  |
| 128 | C128Y | | 82.48% | | C128Y | 42.15% |  |  |  |  | C128Y | 9.91% |
| 129 |  | |  | | A129S | 9.80% |  |  |  |  | A129T | 7.26% |
| 139 |  | |  | |  |  |  |  |  |  |  |  |
| 144 |  | |  | |  |  |  |  |  |  | S144A | 4.62% |
| 153 |  | |  | | D153N | 6.02% |  |  |  |  |  |  |
| 168 |  | |  | |  |  |  |  |  |  |  |  |
| 181 | F181S | | 64.62% | |  |  |  |  |  |  |  |  |
| 184 |  | |  | |  |  |  |  |  |  | P184S | 21% |
| 188 | R188W | | 72.94% | |  |  |  |  |  |  |  |  |
| 190 |  | |  | |  |  |  |  |  |  |  |  |
| 197 |  | |  | |  |  |  |  |  |  | D197A | 5.13% |
| 198 |  | |  | | T198I | 10.56% |  |  |  |  |  |  |
| 200 |  | |  | | I200V | 12.31% |  |  |  |  |  |  |
| 204 | V204F | | 63.18% | |  |  |  |  |  |  |  |  |
| 206 | A206T | | 94.22% | |  |  |  |  |  |  |  |  |
| 206 | A206T | | 94.83% | |  |  |  |  |  |  |  |  |
| 206 | A206T | | 68.01% | |  |  |  |  |  |  |  |  |
| 206 | A206T | | 37.79% | |  |  |  |  |  |  |  |  |
| 207 | W207R | | 93.36% | |  |  |  |  |  |  |  |  |
| 209 |  | |  | |  |  |  |  |  |  |  |  |
| 210 | A210D | | 93.53% | |  |  |  |  |  |  |  |  |
| 210 | A210S | | 95.15% | |  |  |  |  |  |  |  |  |
| 210 | A210T | | 38.72% | |  |  |  |  |  |  |  |  |
| 210 | A210T | | 62.68% | |  |  | A210T | 10.33% |  |  |  |  |
| 210 | A210T | | 17.96% | |  |  | A210T | 10.60% |  |  |  |  |
| 210 |  | |  | |  |  |  |  |  |  |  |  |
| 210 |  | |  | |  |  |  |  |  |  |  |  |
| 216 | D216Y | | 90.72% | |  |  |  |  |  |  |  |  |
| 216 | D216A | | 21.02% | |  |  |  |  |  |  |  |  |
| 220 | L220P | | 76.98% | |  |  |  |  |  |  |  |  |
| 229 |  | |  | |  |  | D229G | 13.80% |  |  |  |  |
| 232 |  | |  | |  |  | L232R | 11.39% |  |  |  |  |
| 234 | A234D | | 7.50% | |  |  |  |  | A234T | 70.37% |  |  |
| 234 |  | |  | |  |  |  |  |  |  |  |  |
| 257 |  | |  | | T257N | 15.46% |  |  |  |  |  |  |
| 260 |  | |  | | A260D | 6.80% |  |  |  |  |  |  |
| 260 |  | |  | | A260D | 10.88% |  |  |  |  |  |  |
| 266 | A266T | | 6.60% | |  |  |  |  |  |  |  |  |
| 268 |  | |  | |  |  |  |  |  |  | L268F | 55.80% |
| 268 |  | |  | |  |  |  |  |  |  | L268F | 25.57% |
| 276 |  | |  | | M276R | 7.20% |  |  |  |  |  |  |
| 277 |  | |  | | N277K | 12.02% |  |  |  |  |  |  |
| 281 | I281R | | 38.85% | |  |  |  |  |  |  |  |  |
| 282 |  | |  | |  |  |  |  | L282S | 7.00% |  |  |
| 289 | D289T | | 49.75% | |  |  |  |  |  |  |  |  |
| 289 | D289T | | 22.03% | |  |  |  |  |  |  |  |  |
| 292 | T292K | | 61.08% | |  |  |  |  |  |  |  |  |
| 295 | D295V | | 60.45% | |  |  |  |  |  |  |  |  |
| 296 |  | |  | |  |  |  |  |  |  |  |  |
| 296 |  | |  | |  |  |  |  |  |  | V296F | 66% |
| 299 | Q299R | | 55.62% | |  |  |  |  |  |  | Q299R | 23% |
| 305 |  | |  | |  |  |  |  |  |  |  |  |
| (+3) |  | |  | |  |  |  |  |  |  |  |  |
| (+4) |  | |  | |  |  | K(+4)T | 9.58% |  |  |  |  |

**S4 Table. Retrieved mutations from selection experiments and Nanopore sequencing coverage in percentage (%)**. Table displaying all the retrieved mutations from different VSV-M^pro^ variants used: VSV-O/A206T-M^pro^, VSV-Omicron-M^pro^, VSV-L167F-M^pro^, VSV-L167F/P168S-M^pro^ and VSV-L167F/F305L-M^pro^. For each substitution, the coverage (in percentage) is reported on its right.
