# Supplementary material for: A comprehensive study of SARS-CoV-2 main protease (Mpro) inhibitor-resistant mutants selected in a VSV-based system
Source: PLoS Pathog. 2024 Sep 11;20(9):e1012522. doi: 10.1371/journal.ppat.1012522 (PMC11407635; doi:10.1371/journal.ppat.1012522)
Supplement: Attachment — Submitted filename: 20240627 Revision Plos Pathogens.docx [file ppat.1012522.s025.docx]

**Reviewer #1**: In this work, the authors use a previously described VSV system to identify resistance mutations that may arise in SARS-CoV-2 upon passage in protease inhibitors. They propose that the use of this system may avoid the gain-of-function concerns associated with performing these types of studies directly in SARS-CoV-2. Compared to the previous work, this work was done with an Omicron NSP5 and an already partially resistant L167F mutant. Further, some additional variants with a number of mutations were selected for further passaging. They then test the effects of the identified mutations in a variety of assays including VSV replication assays as well as biochemical and in cell protease assays. The also perform a number of computational experiments to understand why these variants likely affect protease activity.

**Reviewer #2**: In this manuscript, the authors elaborate on a Vesicular Stomatitis Virus (VSV) model they previously established to investigate the evolution of Mpro mutations resistant to protease inhibitors. Given that Mpro is a key antiviral target, the study of drug resistance mechanisms remains to be an important topic, particularly as SARS-CoV-2 infections persist post-pandemic. The manuscript highlights a congruence (although there are clear differences) between mutations identified in their system and those observed in in vitro studies of SARS-CoV-2, suggesting that their BSL-2 model could provide valuable insights into drug resistance pathways with reduced biohazard risks. Utilizing two initial strains, one expressing the Wuhan Mpro with the L167F drug resistant mutation and the other expressing Omicron Mpro with the P132H mutation, the authors document a distinct mutation trajectory conferring resistance, dependent on the initial Mpro variant. They report variations in resistance levels, with some mutations exhibiting heightened resistance to ensitrelvir compared to nirmatrelvir. To explore the underlying mechanisms, the authors employ computational modeling techniques, including Bioluminate and thermal titration molecular dynamics, to predict alterations in the structural stability of Mpro-nirmatrelvir complexes.

The manuscript is well-written, with solid experimental design and most conclusions are well-supported. However, it lacks a thorough mechanistic characterization of the Mpro mutants (e.g. missing standard enzyme kinetics) and does not fully explore the reasons behind the differing mutation spectrum arising from the Omicron Mpro versus the Wuhan Mpro. The authors also leave out important information about how nirmatrelvir differs from ensitrelvir and how these differences may lead to unique resistance mechanisms. These areas require further elaboration prior to publication as they are, in this reviewer’s opinion, the significant advances this work provides beyond several other reports including their own that identify drug resistant Mpro mutations.

**Reviewer #3**: The manuscript titled “A comprehensive study of SARS-CoV-2 main protease (Mpro) inhibitor-resistant mutants selected in a VSV-based system” by Costacurta et al presents new information on potential Nirmatrelvir resistant mutants using a VSV virus SARS-CoV-2 Mpro inhibitor-resistant mutant were selected using, VSV-Mpro, was applied to produce resistant mutants of nirmatrelvir and ensitrelvir safely and productively. Two live-cell-based protease mutation characterization assays were introduced to quantify the resistance phenotype of mutants. As expected, residues in catalytic site showed up infrequently, and the most frequent mutants were observed in positions further away from the active site. Some of the interesting mutants were thoroughly characterized, and results provide detailed results to better understand the mechanism of the interaction between the protease and inhibitors.

This prediction of resistant mutants of current FDA approved drug, such as nirmatrelvir (Paxlovid), will likely be important for new drug discovery. However, due to the limitations of VSV-Mpro system, the resistant mutants need to be confirmed by biochemical assay and even cell-based assay with authentic SARS-CoV2 mutant. However, this work goes beyond the scope of this paper. This paper is well written and should be accepted.

**Part II – Major Issues: Key Experiments Required for Acceptance**

We thank the reviewers for their detailed reading and interest in our work. Their comments are shown in cursive font and our answers with an indent. We divided longer paragraphs into subsections to address what we understood as separate questions or demands.

Reviewer #1:

*Overall, the paper addresses an important topic as the potential emergence of drug resistance threatens our current antiviral therapies. The system used to identify mutations is clever, but it was described in a previous study. Thus, the impact of this paper is, for the most part, limited to the impact of the mutations identified in the serial passaging experiments. I have one major concern with this part of the study; the NSP5 variants identified were not tested in the context of authentic CoV replication. While I understand the reluctance to generate drug resistant mutations, especially in the omicron genetic background, these types of studies can be done in other ways (such as a non-infectious RNA replicon system). This type of analysis is critical, as the true importance of many of the identified mutants for the field currently remain unclear because the VSV system clearly selects for variants that are specific for that assay system.*

We agree that confirming mutations selected in VSV with gold standard methods is a priority to increase the impact of this work. We therefore tested as many of our mutants as we could achieve with a previously described non-infectious SARS-CoV-2 replicon system. We found that all of the mutations we selected in VSV also confer resistance in the replicon system and that the extend of the resistance mostly correlates when comparing the two systems. We generated a new **Figure 4**, where we describe the replicon system, show the resistance phenotypes of the mutants and correlate them with VSV tested mutations. We describe the **results** of the replicons on **page 14**, **lines 16-25**.

Reviewer #2:

*1. The reliance on theoretical models for insights into mutant vs wild-type Mpro structures, without performing well-established assays to measure enzyme kinetics, significantly weakens the impact of the manuscript.*

In the revised version of this manuscript, we now also include SARS-CoV-2 replicon data in **Figure 4** as well as enzymatic data in **Figure S11** and **Table 1**. Especially the replicon data is a strong approximation of live SARS-CoV-2. The confirmed resistance in the replicons significantly strengthens the mutational data we selected in VSV and this particular approach to avoid gain-of-function research in general.

*1.1. The modeling results are inadequate replacements for empirical data that can provide definitive quantitative measures of Ki, Km, kcat, and catalytic efficiencies. Conducting these measurements for the wild-type and mutants of Mpro (e.g. the mutants they analyzed by computational methods but also the key Omicron mutants), would lend credibility to their models. Specifically, measuring Km’s for model substrates, assessing the catalytic efficiency of the mutants relative to the wild-type, and Ki’s for nirmatrelvir and ensitrelvir will offer important insights into the mechanisms of resistance and structure-function relationships.*

In the revised manuscript in **Figure S11** and **Table 1**, we added dose response experiments of purified Omicron M^pro^ and two of its mutants to the previous wild type M^pro^ and its mutants. Furthermore, we performed kinetic experiments with these seven purified main proteases to generate K_m_ and k_cat_ values. Mutants of Wuhan-1 and Omicron M^pro^ show a lower catalytic efficiency as exhibited by the ratio of k_cat_ to K_m_, indicating a weaker net output than the parental enzymes.

The affinity of nirmatrelvir for M^pro^ is in the high pico- to nanomolar range. In order to measure K_i_ of nirmatrelvir to wild type and mutant variants of M^pro^ we would need to work at a substrate concentration in a similar order of magnitude. This would be possible using high sensitivity detection e.g. using a fluorescence detector and an accordingly labeled substrate peptide. However, the HPLC system we used to determine Michaelis Menten kinetics in the absence of inhibitory compounds does not include such a detector and thus, our detection limit was in the low micromolar range. Therefore, to test the response of selected mutant variants of M^pro^ to nirmatrelvir, we performed dose-response experiments instead to calculate IC_50_ values. We hope our presented results are convincing and support our hypotheses sufficiently.

*1.2. For example, such data will shed light on the differences in substrate binding and inhibitor resistance, providing more clarity on why mutations such as L167F/P168S/L57F confer greater resistance to ensitrelvir compared to nirmatrelvir and how Omicron Mpro derived mutants differ from the Wuhan in its ability to maintain much of the normal proteolytic activity (inferred from their replication assays showing that Omicron derived mutants maintain nearly the same TM50 as the parental strain – Fig.3D).*

Dose response experiments with the purified M^pro^ mutant L167F/P168S/L57F in fact reflect the resistance we detected with VSV-based methods. Although we are unsure how to gain mechanistic insight beyond this confirmation, the modelling of L57F as well as its location in the M^pro^ structure near the S1’ site strongly suggest that its slightly stronger resistance against ensitrelvir can be explained by the binding of ensitrelvir closer to S1’ than nirmatrelvir.

The signature mutation of Omicron, P132H, has been implicated in increased flexibility of the enzyme (PMID: 35292745). Although we would speculate that P132H changes M_pro_’s ability to acquire additional mutations, we did not perform sufficient experiments to confidently make this claim. We produced Omicron M^pro^ and two of its mutants to gain understanding of the difference caused by P132H. The two mutations (A206T and T198I) we tested in purified enzyme retained similar susceptibility to inhibitors as the parental Omicron M^pro^. We also observed this peculiarity of non-resistant Omicron mutants in the On assay. In this assay as well as in VSV-M^pro^, both nsp4/5 and nsp5/6 are processed, whereas in Off, only nsp5/6 is important for the functional read-out. We did not observe resistances in the Omicron On mutant testing, but in Off. In the biochemical assay, where a nsp4/5 cleavage sequence is provided in trans, we also did not observe resistance in the Omicron mutants. The nsp4/5 cleavage site was described to have the highest affinity and fastest kinetic compared to the ten additional M^pro^ cleavage sites in the polyprotein. Since we only observed the non-resistance in Omicron-On and biochemical assays, there is likely a connection of the high-affinity nsp4/5 and P132H influence on the M^pro^ processing. Unfortunately, however, we were not able to elucidate this interesting phenomenon mechanistically.

*1.3. Although in the “Discussion” the authors suggest that individual mutations like L167F, P168S, S144A, and P184S could alter the binding pocket and reduce electrostatic interactions with nirmatrelvir, the specific effects on ensitrelvir binding is not discussed.*

We thank the reviewer for the useful feedback. We investigated the potential common effect of these mutations, which are located near or in the domain II-III linker, and added our observations on **page 19**, **lines 1-9** and **page 22**, **lines 22-32** and **Figure 5D**. We then proceeded to evaluate the effect of mutations L167F, P168S, S144A, and P184S in the combinations they occurred in VSV selection studies on ensitrelvir binding through TTMD simulations. Results are summarized in **Table 2**.

*1.4. Empirical enzymatic analyses will be crucial for understanding these interactions. The authors already show they are able to express and purify the respective mutants of Mpro so preforming the enzymatic assays is highly feasible.*

Unfortunately, the expertise to produce recombinant enzymes was not available in the institute perpetuating the current study. Since purified main proteases were kindly provided by collaborators, we could only ask for a reasonable effort and were grateful to receive several enzymes to verify some of our findings from VSV-based assays.

*2. The authors do not address the distinction between secondary and tertiary mutations that emerge in the Omicron Mpro with the P132H mutation versus those in the Wuhan Mpro.*

We indeed observed that some mutations seemed to occur preferentially in Omicron over Wuhan-1 M^pro^ (such as A206T) and that some of the to-be-mutated residues even interacted with H132 (P132H being the signature mutation of Omicron), for example T198 (becoming T198I). However, we were not confident that we sampled the evolutionary space of M^pro^ sufficiently to draw conclusions from those slightly different signatures. Such a study would require the generation of thousands of mutations to cover the entire sequence of M^pro^ with a depth that is beyond the scope of this project. Instead, we steered the evolution of mutations by selecting those, which were interesting according to the criteria we describe in the **discussion**, **page 25**, **lines 9-17**.

*2.1. Furthermore, the authors have not explored the mechanistic underpinnings that could account for the observed differences in mutation-induced resistance to protease inhibitors. Elucidating how initial mutations can restrict the evolutionary trajectory of Mpro is of substantial interest, offering potential strategies to anticipate and counteract drug resistance. Applying the authors’ modeling approach to address this question would enhance the impact of this work.*

Evaluating the evolutionary trajectory of M^pro^ in response to initial mutations is beyond the scope of the manuscript and the sensitivity of the computational approaches used in the paper. As described above, we also experimentally influenced the development of additional mutations by only focusing and following up several. We therefore cannot claim an unguided development of mutant lines that would likely be more useful to inform broad evolutionary tendencies.

Recent literature however (among others PMID: 35982678) as well as our own results suggest that the evolution in the context of inhibitor pressure can be restricted. One scenario is that initially one mutation with a strong effect on resistance and stability, such as E166V, comes up and compensatory mutations such as T21I restore the activity. A second scenario can be that an initial mutation with less resistance, but also a milder negative effect on stability is selected, such as L167F. When the inhibitor pressure persists, additional mutations are added to lead to a more incremental increase in resistance. The end result in both scenarios is an enzyme that is resistant and stable enough to support viral replication, albeit some attenuation of the virus is still likely.

*2.2. Performing enzymatic assays as suggested in (1) above will also provide needed insight. At a minimum, an analysis of mutants that are unique and only found to occur with the initial variant of Mpro versus those that are found in common would go a long way and perhaps shed important insight into the structural constraints of Mpro.*

Unfortunately, we did not test enough purified M^pro^ mutants to gain insight into the structural constraints of the enzyme. Others have previously focused on this specific topic and published far more sophisticated methods and results to gain such insights. It has been described that M^pro^ is in general more restrained and conserved than other parts of the viral proteome and there are useful online resources (<https://jbloomlab.github.io/SARS2-mut-fitness/>), where one can assess that the penalty for mutations in M^pro^ is higher than for example in Spike.

Comparing SARS-CoV-2 to previous Coronaviruses also illustrates this point. SARS-CoV-2 M^pro^ and SARS-CoV M^pro^ share 96 % sequence identity, whereas their Spikes are only 76 % identical. The strong conservation is remarkable given the fact that those two viruses arose ten years apart. As for Wuhan-1 M^pro^, high-throughput mutational scanning in yeast before the surge of the Omicron variant (PMID: 35723575) revealed that most residues are structurally constrained, leaving only several hotspots for mutations.

However, as mentioned above, the signature mutation of Omicron, P132H, has been implicated in increased flexibility of the enzyme (PMID: 35292745). Although we did not generate enough data with purified enzymes, the results stemming from our M^pro^-Off assay and resulting TM_50_ values would indicate that Omicron M^pro^ is indeed more tolerant in general for additional mutations. The limited resistance and kinetic data we obtained from purified enzymes suggests that the VSV-based assays are capable of generating reliable read-outs, and we therefore think that the Omicron TM_50_ data is therefore a potential proxy for the different structural constraints of Wuhan-1 and Omicron main proteases.

*3. The computational models should include predictions for natural substrates (e.g. peptides that represent NSP4-5 junction) of Mpro. The authors can apply their modeling studies of resistant mutants to determine how binding to substrate might be altered compared to the WT or the initial mutant (e.g. L167F vs Omicron/207). Such information for example is important in their discussion (page 26, lines 5-14) describing the correlation between stability and replication rate – you can have a stable Mpro that nonetheless may have low proteolytic activity and therefore result in slower replication – substrate binding stability will be a better measure to correlate to the TM50’s.*

We investigated the stability of the natural substrate NSP5|6 through static (Bioluminate) and NSP4|5 in dynamic stability modelling (TTMD). In static modelling of NSP5|6, we detected a correlated destabilization of inhibitor and natural substrate (**Fig. S11E**). However, we did not observe meaningful stability differences between the mutants and the wildtype in NSP4|5 dynamic modelling (table below).

| **Model** | **RUN 1** | **RUN 2** | **RUN 3** | **RUN 4** | **RUN 5** | **Mean** |
| --- | --- | --- | --- | --- | --- | --- |
| Wildtype | 0.00375 | 0.00308 | 0.00426 | 0.00461 | 0.00532 | 0.00421 |
| L167F_P168S_L57F | 0.00420 | 0.00492 | 0.00396 | 0.00350 | 0.00335 | 0.00389 |
| L167F_F305L_P184S | 0.00433 | 0.00376 | 0.00397 | 0.00641 | 0.00404 | 0.00411 |
| L167F_F305L_P184S_T21I | 0.00388 | 0.00679 | 0.00412 | 0.00380 | 0.00366 | 0.00393 |

Importantly, TTMD has been initially conceived for investigating protein-small molecule complexes and the applicability of the technique to protein-peptide simulations has not been thoroughly assessed yet. For this reason, we decided not to include this data in the manuscript. Currently, attempts at validating the TTMD methodology for the study of protein-peptide / protein-protein complexes are ongoing in the lab of our molecular dynamic’s collaborator, similarly to what has already been carried out for RNA-small molecule complexes (PMID: 38028536).

Reviewer #3: (No Response)

**Part III – Minor Issues: Editorial and Data Presentation Modifications**

Please use this section for editorial suggestions as well as relatively minor modifications of existing data that would enhance clarity.

**Reviewer #1**:

*In figures 3 and 4, no statistical tests are performed which make it difficult to understand which changes are likely to be meaningful.*

We agree that leaving out information about replicates was careless and could be viewed negatively by the reader. We therefore added standard deviations and replicates to the plots in **figures 3** and **4**. We also performed a Pearson’s correlation (**Figure 4D**) to assess the extent of which the newly generated replicon resistance data corresponds to the data in VSV.

Reviewer #2:

*1. 10-100uM of nirmatrelvir seems to be very high concentrations of drug to use in their experiments. Is expression of Mpro high in their cellular system? Is the difference due to the lack of using efflux inhibitors? Please comment on this in the discussion and in Methods. Could this also contribute to not picking up the T21I mutant?*

We realize that not discussing this difference was a shortcoming of the manuscript. In fact, VSV is among the fastest replicating viruses and its turnover time vastly exceeds that of SARS-CoV-2. Therefore, VSV-M^pro^ produces more M^pro^ in shorter periods than SARS-CoV-2, which requires higher doses of the inhibitor to block the enzyme. We added this explanation to the **discussion** section ‘**caveats of the study**’, **page 29**, **lines 19-23**.

*2. It would be helpful in figure 1 or 2 to include a diagram indicating the dose of drug used for at each passage and the total passages. In Fig. 2B, indicate at what passage number and concentration of nirmatrelvir these mutants were obtained.*

In the revised version of the manuscript, we included a detailed scheme of the passaging and concentrations in **supplemental Figure 2A and supplemental Table 1**.

*3. The mutants with >500 entries would be more informative if the authors included which CoV-2 variant they might be associated with (e.g. alpha, delta, omicron, etc) – if that information is available.*

To include this information, we added a new **supplemental Table 2** detailing in which variants of concern the above 500 mutations occurred most frequently.

*4. Nirmatrelvir and ensitrelvir inhibit Mpro by distinct mechanisms (as reported in Ref 42 – Noske et al. JBC 2023). Ensitrelvir is a nonpeptide noncovalent inhibitor and nirmatrelvir is a covalent peptidomimetic inhibitor. There should be some discussion about the different mechanisms of inhibition. For example, how might the mechanism of inhibition explain the difference in resistance to either drug displayed by L167F/P168S/L57F (Fig. 4D)?*

As the reviewer suggested, we added a panel (**Fig. 6A**) showing the different binding of nirmatrelvir and ensitrelvir to the catalytic pocket of M^pro^. We also describe in more detail the potential reasons for the different effects of L167F/P168S/L57F on **page 19**, **lines 1-9** and **page 22**, **lines 22-32**. The stronger resistance to ensitrelvir was also confirmed by testing L57F, L167/P168S and L167/P168S/L57F with purified M^pro^ (**Table 1**).

Reviewer #3: (No Response)
